# Imputation of missing genotypes: an empirical evaluation of IMPUTE

**DOI:** 10.1186/1471-2156-9-85

**Published:** 2008-12-12

**Authors:** Zhenming Zhao, Nadia Timofeev, Stephen W Hartley, David HK Chui, Supan Fucharoen, Thomas T Perls, Martin H Steinberg, Clinton T Baldwin, Paola Sebastiani

**Affiliations:** 1 Department of Biostatistics, Boston University School of Public Health, 801 Massachusetts Avenue, Boston MA 02118, USA; 2 Department of Medicine, Boston University School of Medicine, 72 East Concord Street, Boston MA 02118, USA; 3 Centre for Research and Development, Medical Diagnostic Laboratories, Faculty of Associated Medical Sciences, Khon Kaen University, Khon Kaen, 40002, Thailand; 4 Geriatric Section, Boston Medical Center, Boston 02118 MA, USA

## Abstract

**Background:**

Imputation of missing genotypes is becoming a very popular solution for synchronizing genotype data collected with different microarray platforms but the effect of ethnic background, subject ascertainment, and amount of missing data on the accuracy of imputation are not well understood.

**Results:**

We evaluated the accuracy of the program IMPUTE to generate the genotype data of partially or fully untyped single nucleotide polymorphisms (SNPs). The program uses a model-based approach to imputation that reconstructs the genotype distribution given a set of referent haplotypes and the observed data, and uses this distribution to compute the marginal probability of each missing genotype for each individual subject that is used to impute the missing data. We assembled genome-wide data from five different studies and three different ethnic groups comprising Caucasians, African Americans and Asians. We randomly removed genotype data and then compared the observed genotypes with those generated by IMPUTE. Our analysis shows 97% median accuracy in Caucasian subjects when less than 10% of the SNPs are untyped and missing genotypes are accepted regardless of their posterior probability. The median accuracy increases to 99% when we require 0.95 minimum posterior probability for an imputed genotype to be acceptable. The accuracy decreases to 86% or 94% when subjects are African Americans or Asians. We propose a strategy to improve the accuracy by leveraging the level of admixture in African Americans.

**Conclusion:**

Our analysis suggests that IMPUTE is very accurate in samples of Caucasians origin, it is slightly less accurate in samples of Asians background, but substantially less accurate in samples of admixed background such as African Americans. Sample size and ascertainment do not seem to affect the accuracy of imputation.

## Background

Missing genotype data in genetic association studies is a common problem often caused by poor DNA quality and inadequate genotype calling algorithms [1], and imputation has been widely used to infer missing genotype data [2]. Strategies for imputation that are specific to genetic data leverage knowledge of linkage disequilibrium (LD) between single nucleotide polymorphisms (SNP) to reconstruct haplotypes that are used to inform imputation. The most popular solution is implemented in fastPHASE [3], that uses a Hidden Markov model to describe the spatial distribution of clusters of haplotypes along a chromosome and reconstructs individual haplotypes from unphased genotype data using a Bayesian rule. Machine learning methods that use k-nearest-neighbor, classification and regression trees, or Bayesian networks have also been proposed to impute missing genotype data in relatively small datasets and were evaluated in [4,5] and [6]. Sun and Kardia [7] have recently proposed a neural-network based approach and, although computationally more efficient, none of these alternative methods was able to reach the high accuracy of fastPHASE under a variety of conditions [5,7].

Marchini et al adapted this model from haplotype to genotype data and implemented it in the software IMPUTE [1]. The algorithm in IMPUTE models the probability of the vector of genotypes *G*_*i *_= {*G*_*i*1_,...,*G*_*iL*_} at the L loci of subject *i*, given a set of known haplotypes H, using a Hidden Markov model with hidden states that represent pairs of haplotypes from the set H. The key feature of this method is the use of the information from all markers in LD with the SNPs to be imputed in order to infer the missing genotypes. The set of known haplotypes can be derived from publicly available data such as that created by the International HapMap project [8]. The great potential of this method is to allow investigators to synchronize genotype data that were typed using different platforms and several authors have shown that this approach increases the power of genome-wide association studies [9].

This strategy is now widely accepted and genome wide association studies that include in their analysis imputed genotype data of untyped SNPs are becoming very common [10-14]. However, the original report [1] presented results of an evaluation based on control data from the Welcome Trust Case Control Consortium [10] and focused mainly on SNPs typed with the Affymetrix 500 K array in a cohort of Caucasian subjects. Because subject ascertainment, and differences in the genetic background of study subjects and in the design of the platforms may influence the accuracy of the imputation, we decided to extend the original evaluation to include populations with different genetic backgrounds and cases of rare disease.

## Methods

We used publicly available genotype data from a US Caucasian population of 270 neurologically normal controls (NNC) used in [15], an African American population of 111 sickle cell anemia patients (SCA) enrolled in the Multicenter Study of Hydroxyurea [16], a US Caucasian population of 280 centenarians enrolled in the New England Centenarian Study [17] (NECS), an African American population of 258 random controls (AA) extracted from the Illumina genotype control database , and a Thai population of 104 β-thalassemia carriers (THAI). The first data set combines genotype data from the Illumina Infinium I (human-1) and Infinium II (humanhap300-duo) platforms. The human-1 array has assays of almost 110,000 gene-centric SNPs while the humanhap300-duo array has approximately 317,000 haplotype tagging SNPs that are based on the Phase I of the International HapMap Project [8]. The two arrays represent more than 400,000 unique SNPs. All the other data sets were typed with the Illumina humanCNV 370 array with approximately 350,000 haplotype tagging SNPs selected from Phase I and II of the HapMap project.

Beside their availability, the rationale to use these populations in our evaluation was based on the following observations. The NNC set represents a "referent" Caucasian group that should not be enriched with subjects having a particular disease. We included the NECS set to examine the accuracy of IMPUTE in a Caucasian population with a rare trait that is supposed to be regulated by several genes [18] so that, genetically, these subjects may be substantially different from randomly selected individuals from North America. Similarly, the AA and SCA sets are two different groups of African Americans: the former consists of randomly selected subjects with varied levels of genetic admixture between Africans and Caucasians, while the latter should comprise subjects who are genetically more homogeneous because they are all affected with SCA. This feature should make them closer to Africans [19]. The THAI set consists of subjects with a genetic background that should be different from both the Chinese Han and Japanese panel used in the HapMap project [20]. Therefore, with the exclusion of the NNC set, all the other groups have characteristics that could make them substantially distant from the HapMap panel and impact the accuracy of the imputation.

We used this data to assess the extent of the accuracy claimed in the original manuscript for increasing proportions of missing data, different sample sizes and SNP selection. We started our evaluation using the NNC set (Table 1) that should be the easiest case, and chromosome 21 that is tagged by the smallest number of SNPs (~5900 in the NNC set) compared to the other chromosomes, and removed either 100% or 80% of genotype data in an increasing proportion of randomly selected SNPs. Each simulation was repeated 1,000 times, and in each set we used the program IMPUTE to fill in the missing genotypes using the haplotypes inferred with the HapMap data from Utah residents with ancestry from northern and western Europe (CEU) as the reference population. The default parameters of IMPUTE were used. In each of the 1,000 runs, we computed the proportion of genotypes that were correctly imputed compared to the observed ones and to summarize the results we estimated the final accuracy as the median proportion of correctly imputed genotypes across different runs. We used the same procedure to evaluate the accuracy of IMPUTE in the other datasets and we repeated the analysis using also chromosome 2 that is tagged by the largest set of SNPs (~29,800 in the NNC set). We used IMPUTE with and without splitting this chromosome to 10 Mb chunks. Results are in Tables 2 and 3. We used reference haplotypes from the CEU set to impute genotype data in the NECS, from the Yoruba in Ibadan (YRI) set to impute data in the SCA and AA sets, and we combined the sets of haplotypes from the Japanese in Tokyo, Japan (JPT) and the Han Chinese in Beijing, China (CHB) to impute the data in the THAI set. The effective population sizes we used were: 11418 for CEU, 17469 for YRI and 14269 for CHB/JPT.

**Table 1:** Summary of the accuracies of IMPUTE using data from chromosome 21 in the NNC set

**Accuracy**						
	**Missing**	**0.1%**	**1%**	**10%**	**40%**	**60%**

**Complete missing**	**Overall**	97.42% (0.20, 0.93, 0.99, 1)	97.42% (0.01, 0.93, 0.99, 1)	97.05% (0.01, 0.92, 0.99, 1)	95.20% (0.01, 0.88, 0.99, 1)	91.88% (0.01, 0.82, 0.97, 1)
	
	**0.95 P.P.**	99.24% (0.00, 0.98, 1.00, 1)	99.24% (0.00, 0.98, 1.00, 1)	99.22% (0.00, 0.98, 1.00, 1)	99.06% (0.00, 0.98, 1.00, 1)	98.86% (0.00, 0.97, 1.00, 1)
	
	**Percentage**	82.30%	82.31%	80.38%	71.16%	59.39%

**80% missing**	**Overall**	97.24% (0.28, 0.93, 0.99, 1)	97.70% (0.01, 0.93, 0.99, 1)	97.24% (0.01, 0.93, 0.99, 1)	95.39% (0.01, 0.88, 0.99, 1)	91.71% (0.00, 0.82, 0.97, 1)
	
	**0.95 P.P.**	99.38% (0.00, 0.98, 1.00, 1)	99.42% (0.00, 0.98, 1.00, 1)	99.27% (0.00, 0.98, 1.00, 1)	99.04% (0.00, 0.98, 1.00, 1)	98.95% (0.00, 0.97, 1.00, 1)
	
	**Percentage**	81.92%	82.08%	80.53%	71.39%	59.12%

The columns report the accuracy of imputation when different proportions of SNPs ranging from 0.1% to 60% were imputed. The first three rows labelled as "Complete missing" summarize the accuracy when the genotype data were completely removed, while the last three rows labelled "80% missing" summarize the accuracy when 80% of the genotype data were randomly removed. The row labelled "Overall" reports the median accuracy and the minimum, 1^st ^quartile, 3^rd ^quartile, and maximum accuracy value within brackets. The row labelled "0.95.P.P" reports the median accuracy of the imputed genotypes when a minimum posterior probability of 0.95 was required for an imputed genotype to be acceptable. The row labelled "Percentage" reports the percentage of imputed genotype data that were acceptable by using the minimum posterior probability of 0.95 as a requirement.

**Table 2:** Impact on imputation accuracy of splitting chromosomes into chunks

**Accuracy**					
		**NNC (split)**	**NNC (non split)**	**SCA (split)**	**SCA (non split)**

**Complete missing**	**Overall**	97.42% (0.01, 0.93, 0.99, 1)	97.42% (0.01, 0.93, 0.99, 1)	88.29% (0.01, 0.79, 0.95, 1)	88.29% (0.01, 0.79, 0.95, 1)
	
	**0.95 P.P.**	99.23% (0, 0.98, 1, 1)	99.23% (0.00, 0.98, 1.00, 1)	97.30% (0.00, 0.94, 1.00, 1)	97.30% (0.00, 0.94, 1.00, 1)

**80% missing**	**Overall**	97.24% (0.01, 0.93, 0.99, 1)	97.70% (0.01, 0.93, 0.99, 1)	88.76% (0.01, 0.80, 0.96, 1)	88.76% (0.01, 0.80, 0.96, 1)
	
	**0.95 P.P.**	99.28% (0.00, 0.98, 1.00, 1)	99.30% (0.00, 0.98, 1.00, 1)	97.44% (0.00, 0.94, 1.00, 1)	97.44% (0.00, 0.94, 1.00, 1)

No obvious impact of splitting chromosome 2 into small chunks of 10 Mb on imputation accuracy while using the data from the NNC and SCA sets. In all tests, 10% of the SNPs on chromosome 2 were randomly selected and their genotype data were either completely removed (Complete missing), or only 80% randomly removed (80% missing).

**Table 3:** Comparison of the accuracies of the imputed genotypes in different populations

**Accuracy**							
**Population (sample size)**		**AA (258)**	**SCA (111)**	**THAI (104)**	**NNC (270)**	**NNC (135)**	**NECS (280)**

**Complete missing**	**Overall**	85.66% (0.26,0.75,0.93,1)	87.39% (0.02,0.77,0.95,1)	94.23% (0.01,0.86,0.98,1)	97.05% (0.01,0.92,0.99,1)	97.06% (0.02,0.93,0.99,1)	96.43% (0.01,0.91,0.99,1)
	
	**0.95 P.P.**	96.70% (0.00,0.92,0.99,1)	97.22% (0.00,0.94,1.00,1)	98.06% (0.00,0.95,1.00,1)	99.22% (0.00,0.98,1.00,1)	99.24% (0.00,0.98,1.00,1)	99.15% (0.00,0.98,1.00,1)
	
	**Percentage**	59.00%	60.77%	72.56%	80.38%	80.40%	77.43%

**80% missing**	**Overall**	85.92% (0.29,0.75,0.93,1)	87.64% (0.01,0.78,0.94,1)	93.98% (0.01,0.86,0.98,1)	97.24% (0.01,0.93,0.99,1)	97.25% (0.01,0.93,0.99,1)	96.43% (0.01,0.91,0.99,1)
	
	**0.95 P.P.**	96.79% (0.00,0.92,0.99,1)	97.37% (0.00,0.93,1.00,1)	98.46% (0.00,0.95,1.00,1)	99.27% (0.00,0.98,1.00,1)	99.08% (0.00,0.98,1.00,1)	99.08% (0.00,0.98,1.00,1)
	
	**Percentage**	59.03%	61.27%	72.82%	80.53%	80.44%	77.43%

The columns report the accuracy of imputation when 10% of SNPs were imputed. As in Table 1, the first three rows labelled as ''Complete missing'' summarize the accuracy when the genotype data were completely removed, while the last three rows labelled ''80% missing'' summarize the accuracy when 80% of the genotype data were randomly removed. The row labelled ''Overall'' reports the median accuracy and the minimum, 1^st ^quartile, 3^rd ^quartile, and maximum accuracy within brackets. The row labelled ''0.95.P.P'' reports the median accuracy of the imputed genotypes when a minimum posterior probability of 0.95 was required for an imputed genotype to be acceptable. The row labelled ''Percentage'' reports the percentage of imputed genotype data that were acceptable by using the minimum posterior probability of 0.95 as requirement.

## Results and discussion

**Figure 1:**
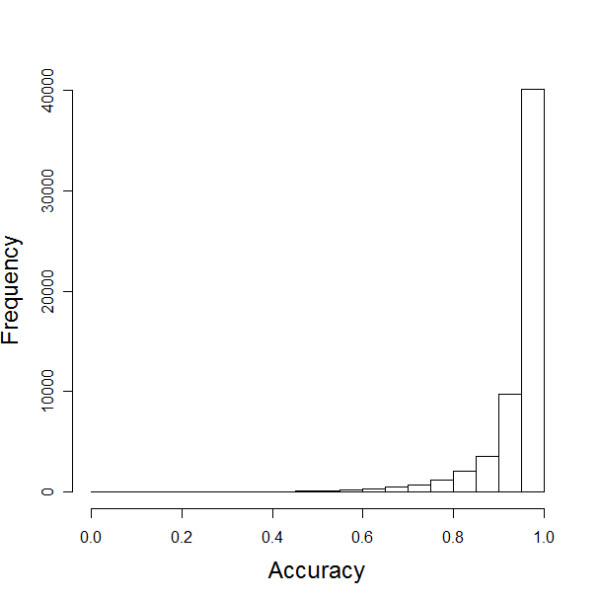
**Distribution of imputation accuracies when 1% of the SNPs were randomly selected from chromosome 21 and their genotype data completely removed in the NNC set.** The results for other proportion of missing SNPs are in the supplementary material. In each of the 1,000 simulations we randomly selected 1% of the SNPs to be removed from the data and their genotype data to be imputed. The chromosome is tagged by approximately 5,900 SNPs, so that 59 SNPs were removed in each run, and 59,000 SNPs had to be imputed across all 1,000 simulations. The x-axis reports the accuracy of each of the 59,000 SNPs that were imputed in the 1,000 simulations. The y-axis reports the frequency of different imputation accuracies.

Table 1 shows the summary statistics of the accuracy of the method when we impute an increasing proportions of SNPs in chromosome 21 in the NNC set. The results confirm a very good accuracy of the imputation method when either 100% or 80% of genotypes are missing in up to 40% of the SNPs. In fact, more than 40% of the SNPs have to be missing to lower the median accuracy to less than 95%. The median accuracy increases to 99% when we impose a posterior probability greater than 0.95 as the threshold to accept the imputed genotypes. This increased accuracy competes with the ability to complete the data as only 71–82% of imputed genotypes were acceptable. Figure 1 shows the distribution of imputation accuracy when 100% of genotypes in 1% randomly selected SNPs were removed in the genotype data of chromosome 21 in the NNC set. The data are essentially those summarized in column 2 of Table 1 and show a clear skewness of the results with a very small number of SNPs that failed to be imputed correctly while the majority of SNPs was imputed with large accuracy. We examined 30 SNPs with very low accuracy and found that most of them are in recombination hotspots which were estimated from Phase II Hapmap data.

**Figure 2:**
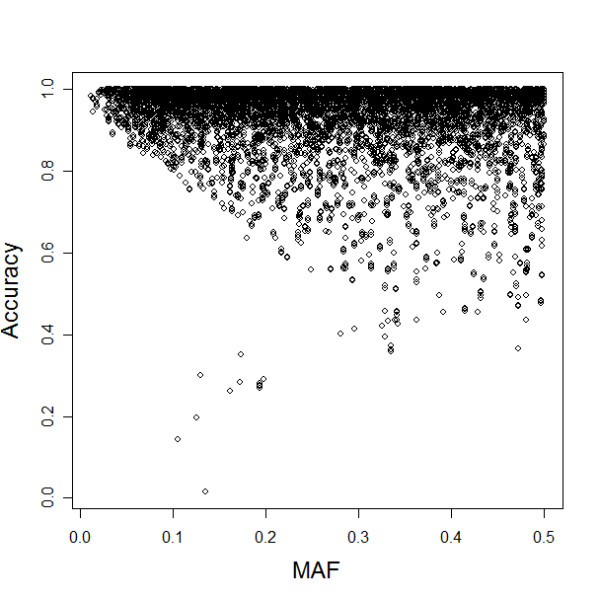
**Accuracies versus minor allele frequency (MAF), when 1% of the SNPs on Chr21 were randomly selected and their genotype data were completely removed in NNC set.** The cluster of 10 points corresponds to SNPs that are in recombination hotspots.

**Figure 3:**
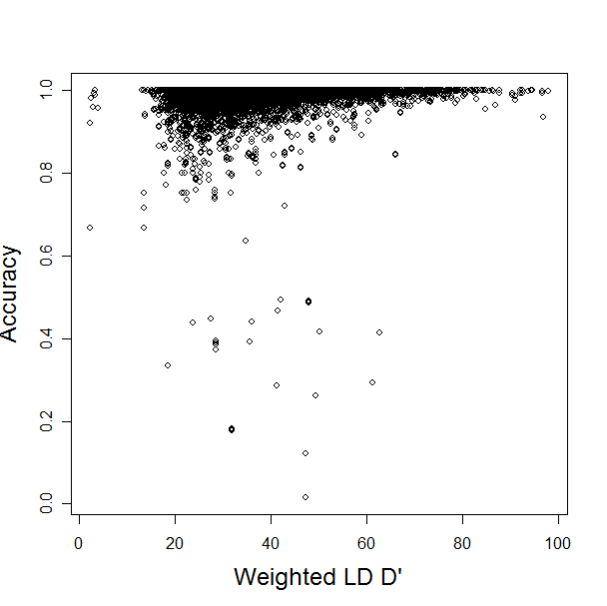
**Accuracies of imputed genotypes in 59,000 SNPs (y axis) versus a summary of the LD patterns surrounding them (x axis).** The summary of LD is a weighted average of the pairwise D' between each SNP to be imputed and all other SNPs in the same chromosome with weights that are calculated as wD'=∑i=1nDi'exp⁡(−di). In the formula, *d*_*i *_is the physical distance between the SNP to be imputed and the ith SNP, in 100 kb, and *d*_*i*_' is the estimate of LD between the same two SNPs.

In addition, Figure 2 shows the accuracy of imputed genotypes (when 1% of the SNPs on Chr21 were randomly selected and their genotype data were completely removed in NNC set) as a function of the SNPs minor allele frequency (MAF) and shows that imputation of SNPs with smaller MAF appears to be more accurate than imputation of the SNPs with larger MAF. This is consistent with our expectation and suggests that imputation of SNPs with almost uniform allele frequencies may not be reliable. We also measured the accuracy of the inferred genotypes as a function of the strength of LD. The plot in Figure 3 shows that, with the exception of a few SNPs that may be recombination hotspots, the accuracy is very high even when the target SNPs are not in strong LD (D' < 0.7) with other SNPs that are used to reconstruct the imputation model.

In the evaluation we chose the two proportions of 40% and 60% SNPS to be fully imputed to create the hypothetical scenario of integrating data from the Affymetrix 500 K and the Illumina 370 K arrays. The two platforms have approximately 53,200 SNPs assayed in common so that the union of SNPs in the two arrays consists of approximately 820 K SNPs, and one needs to impute 60% of the SNP to synchronize Illumina with Affymetrix data (increase from 370 K to 820 K), and 40% of the SNPs to synchronize Affymetrix with Illumina data (increase from 500 K to 820 K). We observed a median accuracy of 95.20% when imputing 40% of the SNPs, while the accuracy goes down to 91.88% when 60% of the SNPs are to be fully imputed. The first case (imputation of 40% of the SNPs) would be close to synchronizing the data generated from the Affymetrix 500 K with those generated with the Illumina 370 K array, while the second case would be close to synchronizing genotype data generated with the Illumina 370 K array with those generated with the Affymetrix 500 K platform. The accuracy slightly improves when at least 20% of the genotype data are known and this data can be used to build the imputation model. The median accuracy increases to 99% when we impose a posterior probability greater than 0.95 as the threshold to accept the imputed genotypes, but this increased accuracy again competes with the ability to complete the data as only 70–80% of imputed genotypes are acceptable. This result would suggests that some caution is needed when trying to synchronize genotype data collected with the Illumina 370 K array with those collected with the Affymetrix 500 K array. However, a serious limitation of our analysis is that we did not consider the fact that Affymetrix and Illumina use different methods to select tagging SNPs and the distribution of SNPs is not uniform on the chromosomes between these two platforms. Therefore, more evaluation is needed to really understand the reliability of synchronizing data from these two platforms.

We did not see significant differences in accuracies between the simulations conducted with data from chromosomes 2 and 21 (Table 2) and this finding suggests that chromosome size and the effective number of SNPs do not interfere with the performance of the method.

Table 3 reports the results of the simulations that we extended to include populations of different genetic backgrounds. Because the initial analysis in the NNC set showed little variations of the accuracy for a wide proportion of SNPs to be imputed, we chose to randomly select only 10% of the SNPs in chromosome 21 and either removed 100% of their genotype data or 80%. Compared to the results in the NNC set, the accuracies of the imputed genotypes in the NECS set are slightly lower, while the accuracies of imputed genotype data in African Americans and Asians are substantially lower (Table 2). When only 10% SNPs are completely missing, the median accuracy of IMPUTE is 85.66% in the data from random African American controls, 87.39% for the SCA set, and 94.23% for Thai samples. The accuracies increase to 96.70%, 97.22% and 98.06% if we require that the posterior probability of the imputed genotypes is at least 0.95, but again this increased precision leaves approximately 20–30% of missing data. Because both the SCA and THAI sets have a smaller sample size compared to the NNC set, we also repeated the simulations in the NNC set using a sample size comparable to the other ethnic groups to remove possible sample size effects. The analysis showed no difference in accuracies thus confirming the conjecture that the lower precision of IMPUTE in the AA and SCA sets is not due to the smaller sample size but may be a consequence of the lower representativeness of the YRI haplotypes. In addition, we tested the effect of chromosome size in SCA set by dividing Chr2 to 10 Mb chunks and did not see any obvious difference in imputation accuracies.

**Figure 4:**
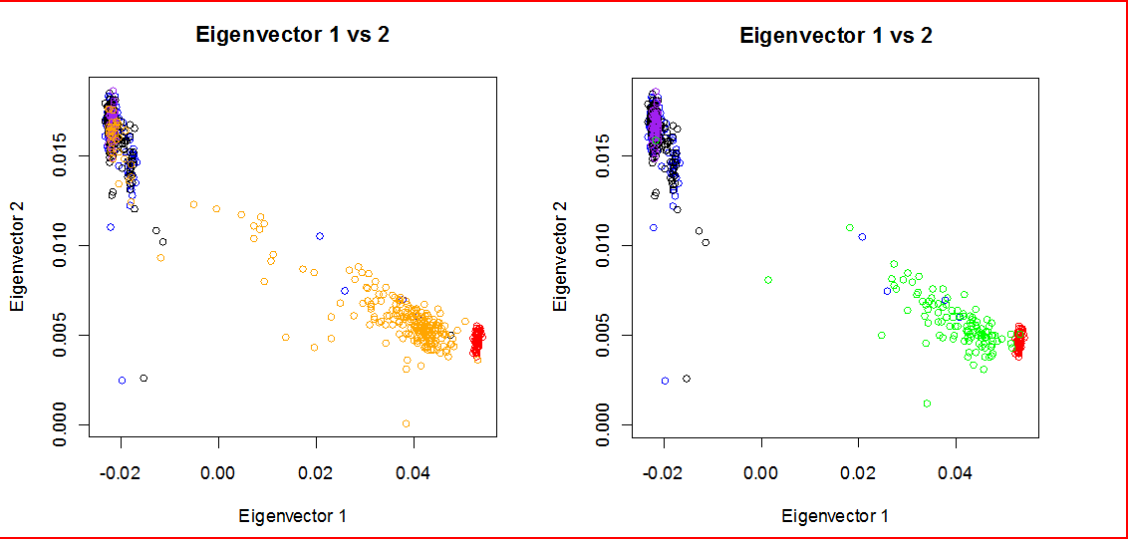
**Results for the principal components analysis (PCA) assessing the degree of stratification between the samples used for imputation and the four Hapmap populations.** The two panels plot the top two principal components for CEU (Purple), YRI (Red), NNC (Black), NECS (Blue), AA (Orange), SCA (Green). The left panel shows that the African Americans (orange) are more admixed as compared to the SCA (green) in the right panel.

Considering that African Americans are genetically a mixture of Africans and Caucasians, we conducted a principal component analysis (PCA) with the EIGENSTRAT program [21] to assess the degree of stratification between the samples used for imputation and the four Hapmap populations [8] (see Figure 4). PCA identifies samples with common ancestry by examining similarities across a large set of SNPs and then assigning similar values for continuous axes of variation to those samples with common ancestry. We found that the Yoruban samples are genetically closer to the SCA samples (Fst = 0.007) when compared with the AA set (Fst = 0.020). Since the Yoruban samples served as the reference population for the imputation of both the SCA and AA sets, it is not surprising that IMPUTE reaches a higher accuracy in the SCA set rather than in the AA set. This observation agrees with the conjecture that African Americans with SCA are less admixed than general African Americans [22]. The analysis also suggests a strategy to increase the imputation accuracies of genotype data from AA samples: one may use the results of PCA to partition the subjects into two clusters based on their similarity to the Caucasian and African populations of the HapMap and then impute the data using as reference haplotypes those of the closest population. We followed this heuristic and split the AA set into two groups of 35 subjects closest to the CEU cluster and 223 subjects closest to the Yoruban. As shown in Table 4, comparing to the original 85.66% accuracy, imputation of genotype data in those subjects who are close to the Yorubans reached an accuracy of 87.88% that is consistent with the results of the SCA set, and the cluster close to the CEU reached an accuracy of 97.14%.

**Table 4:** Accuracy of imputation in samples from African Americans

**Accuracy**				
**Missing**		**Random AA (258)**	**AA close to YRI (223)**	**AA close to CEU (35)**

**Complete missing**	**Overall**	85.66% (0.26, 0.75, 0.93, 1)	87.00% (0.27, 0.77, 0.94, 1)	97.14% (0.11, 0.91, 1.00, 1)
	
	**0.95 P.P.**	96.70% (0.00, 0.92, 0.99, 1)	96.97% (0.00, 0.93, 0.99, 1)	100.00% (0.00, 0.97, 1.00, 1)

**80% missing**	**Overall**	85.92% (0.29, 0.75, 0.93, 1)	87.08% (0.26, 0.77, 0.94, 1)	96.43% (0.07, 0.89, 1.00, 1)
	
	**0.95 P.P.**	96.79% (0.00, 0.92, 0.99, 1)	97.06% (0.00, 0.93, 0.99, 1)	100.00% (0.00, 1.00, 1.00, 1)

Impact of splitting samples from African Americans into groups based on their similarity to the Yorubans and Caucasians on the imputation accuracy. The 1^st ^column reports imputation accuracy when YRI haplotypes are used on the whole set, the 2^nd ^column reports imputation accuracy when YRI haplotypes are used on a cluster of subjects close to YRIs, the 3^rd ^column reports imputation accuracy when CEU haplotypes are used on a cluster of subjects close to CEUs.

The computational speed and memory usage of IMPUTE depend on the sample size and chromosome length. In our cases, imputing 10% of missing SNPs on a small chromosome (such as chr21) for 270 NNC subjects took ~20 min and ~500 MB RAM. For a larger chromosome (chr2) and larger sample (such as 1,000 subjects), we had to divide the chromosomes into small chunks of 10 Mega bases, otherwise it would exceed the maximum memory of common computers.

## Conclusion

The goal of our evaluation was to assess the effect of ethnicity, ascertainment, and different SNP selection on the accuracy of imputation of unobserved SNPs. Our analysis suggests that IMPUTE is very accurate in samples of Caucasian origin, it is slightly less accurate in samples of Asian background, but substantially less accurate in samples of admixed background such as African Americans. The lower accuracy may be an effect of the choice of reference populations and the increasing numbers of control samples that are becoming available to investigators will allow the development of better reference panels and improve the results.

We are currently extending our evaluation to include the program Bim-Bam [9], and MACH 1  that use a similar approach to impute and analyze untyped SNPs. Although we expect the accuracy of imputation to be similar, an open question is to compare the procedures implemented in these different programs to analyze imputed genotype data. We conducted a very preliminary analysis to examine whether a naive analysis of imputed data that ignores the fact that data were imputed inflates the false positive rate and the results suggest that this procedure does not inflate the false positive rate. However, a more comprehensive evaluation is needed.

Although our analysis shows that imputation is feasible even for genome-wide data, an open conjecture is whether the gain of accuracy of IMPUTE, or of similar programs such as fastPHASE, compared to faster but slightly less accurate methods may not be sufficiently large to justify the computational efforts. Machine learning procedures such as KNN and general classification models that we investigated in [4] may require some intelligent search procedure to be applicable to the size of genome-wide data sets but, as we discussed in our earlier work, they have the advantage of using long range LD that can span different chromosomes. More work is however needed to make these alternative procedures applicable to genome wide data.

## Supplementary material

Available at: 

## Authors' contributions

ZZ designed, conducted and analyzed the simulations and prepared a draft of the article. NT conducted the stratification analysis and participated in the manuscript writing. SH provided data management support. DC, SF, TTP, MHS and CB provided access to data for the evaluation, helped to interpret the results and contributed to the manuscript writing. PS conceived and designed the study, coordinated the work, and participated in the interpretation of the results and the manuscript writing. All authors read and approved the final manuscript.
